# Miniscrews position for a tissue bone borne palatal C-expander affects the displacement pattern of nasomaxillary complex: a finite element study

**DOI:** 10.1038/s41598-023-44432-9

**Published:** 2023-10-10

**Authors:** Jin-Young Choi, HyeRan Choo, Min-Jung Kim, Kyu-Rhim Chung, Seong-Hun Kim

**Affiliations:** 1 Department of Orthodontics, Graduate School, Biocreative Orthodontic Strategy (BOS) Center, Kyung Hee University, Seoul, Korea; 2 Division of Plastic and Reconstruction Surgery, Neonatal and Pediatric Craniofacial and Airway Orthodontics, Department of Surgery, Stanford Orthodontic AIrway Plate Treatment Center, Stanford University School of Medicine, Lucile Packard Children’s Hospital, Palo Alto, CA USA

**Keywords:** Health care, Medical research

## Abstract

This study aimed to evaluate the difference in expansion patterns based on the position of miniscrews for a tissue-bone-borne palatal C-expander using a finite element method. Ten expansion models were examined, each representing a different position of miniscrews on the palate. Models A and B had miniscrews symmetrically placed 7 mm and 15 mm below the cementoenamel junction (CEJ), respectively. Models C to J had miniscrews positioned in a triangular manner at 7 mm and 15 mm below CEJ. Stress, displacement, angular changes of the bone and teeth, and changes in the nasomaxillary complex were measured using elastoplastic behavior models through static-nonlinear simulation employing an implicit method. The anterior and posterior parts of paramidpalatal suture area were identified as ANT, TPS-M, and TPS-L, and their ratio was assessed. Model A, which featured three miniscrews located 7 mm below the CEJ, exhibited the least molar inclination and the smallest amount of skeletal expansion. Model I, with two miniscrews placed between the first and second molars, demonstrated the greatest lateral displacement at point N on the nasal cavity wall, along with the smallest ratio of ANT to TPS-M or TPS-L. This finding suggests that the posterior expansion of the palate in relation to the anterior expansion was maximized. The results of this study indicate that strategic positioning of miniscrews is effective in achieving various expansion patterns based on the targeted correction areas within the nasomaxillary complex.

## Introduction

Transverse maxillomandibular discrepancy is a common skeletal malocclusion frequently observed in orthodontic clinics. Typically, this condition arises due to a narrow maxilla caused by inadequate maxillary growth.^1^ A narrow maxilla is associated with a constricted palatal vault, posterior crossbite, and can lead to airway-related issues like nasal airway obstruction and mouth breathing.^2^ Previous studies have shown that maxillary expansion treatment can effectively address these airway manifestations in patients with a narrow maxilla.^3–5^ A conventional palatal expander is capable of widening the nasal region by opening the midpalatal suture in a reverse "V" shape,^6,7^ and this widening of the nasal cavity leads to long-term improvements in airway manifestations.^8^ Moreover, the implementation of a tooth-bone-borne expander, known as miniscrew-assisted rapid palatal expansion (MARPE), has been found to increase airway volume and also enhance muscle strength and oral peak flow.^2^

Different strategies have been employed for orthodontic maxillary arch expansion in patients of varying ages.^9^ Typically, children and adolescents with growth-related narrow maxilla are prescribed wide arch wires, transpalatal arches (TPA), quad helix appliances, or cross-arch elastics to achieve dental expansion. Additionally, tooth-borne rapid palatal expanders or tooth and tissue-borne expanders can be utilized to address transverse deficiencies through orthopedic expansion.^10–12^ However, in skeletally mature adults and late adolescents, the stronger interdigitation of the midpalatal suture presents limitations in resolving transverse deficiencies solely through dental expansion.^13–15^ This can lead to adverse effects on anchor teeth, including tipping, extrusion, root resorption, relapse, and periodontal issues.^16^ Recently, surgical approaches such as surgically assisted rapid palatal expansion (SARPE) or surgical segmental surgery have been introduced for adult patients with maxillary transverse deficiency, and they have shown effectiveness.^17^ However, these surgical methods are invasive and can place a greater burden on patients.^18^ As alternatives to surgical techniques, tooth- and bone-borne expanders, as well as tissue- and bone-borne expanders, have been developed alongside temporary skeletal anchorage devices. Studies have been conducted to assess the effectiveness of these devices.^19–22^.

A specific type of tissue- and bone-borne expander, known as the C-expander, has been introduced and utilized for several decades.^19–22^ Unlike other expansion devices where miniscrews are placed based on the expander design, the C-expander is distinctive in that it involves first strategically placing the miniscrew and then designing the expander body accordingly. Studies have indicated that when compared to tooth-bone-borne palatal expanders, the use of the C-expander results in less molar inclination and buccal alveolar bone loss. This is attributed to the fact that the C-expander does not rely on teeth as anchorage for expansion.^21^.

Due to the complexity of anatomical structures affected by maxillary expansion, accurately predicting the precise changes in adjacent structures after maxillary expansion is nearly impossible. Therefore, the finite element method has been utilized to calculate the amount of stress and displacement following specific procedures. In one particular finite element study, it was demonstrated that the vertical position of miniscrews in the C-expander has an impact on molar inclination. Specifically, a deeper miniscrew position was associated with reduced molar inclination.^23^ However, there have been limited studies investigating the effects of miniscrew positions in tissue- and bone-borne expanders on the three-dimensional changes of adjacent anatomical structures.

Our hypothesis was that the placement of miniscrews would result in varying expansion patterns within the oral and nasomaxillary complexes. Therefore, the objective of this study was to assess the differences in expansion patterns based on the positioning of miniscrews in a tissue and bone-borne palatal expander. This was achieved by calculating the displacement amounts at specific points within the oral and nasomaxillary complexes using a finite element model.

## Results

Table 1 displays the angulation changes of the first and second molars for each experimental model. When all three miniscrews were positioned 15 mm below the CEJ (Model B), the first and second molars exhibited greater inclination compared to when the miniscrews were placed 7 mm below the CEJ (Model A). The vertical position of one out of the three miniscrews, whether placed 7 mm or 15 mm below the CEJ, did not significantly impact the differences in molar inclination, considering the axis tendencies observed in Models C to J. However, the molar inclination varied based on the anteroposterior combination of the miniscrews. When one or more miniscrews were placed between the first and second premolars, the first and second molars tended to be more inclined compared to when no miniscrews were positioned between these premolars. In other words, the molar axes in Models C to F exhibited greater changes compared to those in Models G to J.

**Table 1:** Changes in the axis of upper first molars and second molars after palatal expansion in each model.

	Model A (°)	Model B (°)	Model C (°)	Model D (°)	Model E (°)	Model F (°)	Model G (°)	Model H (°)	Model I (°)	Model J (°)
First molar	0.254	0.363	0.424	0.444	0.447	0.441	0.351	0.305	0.367	0.337
Second molar	0.254	0.367	0.423	0.446	0.449	0.442	0.355	0.311	0.371	0.343

**Figure 1:**
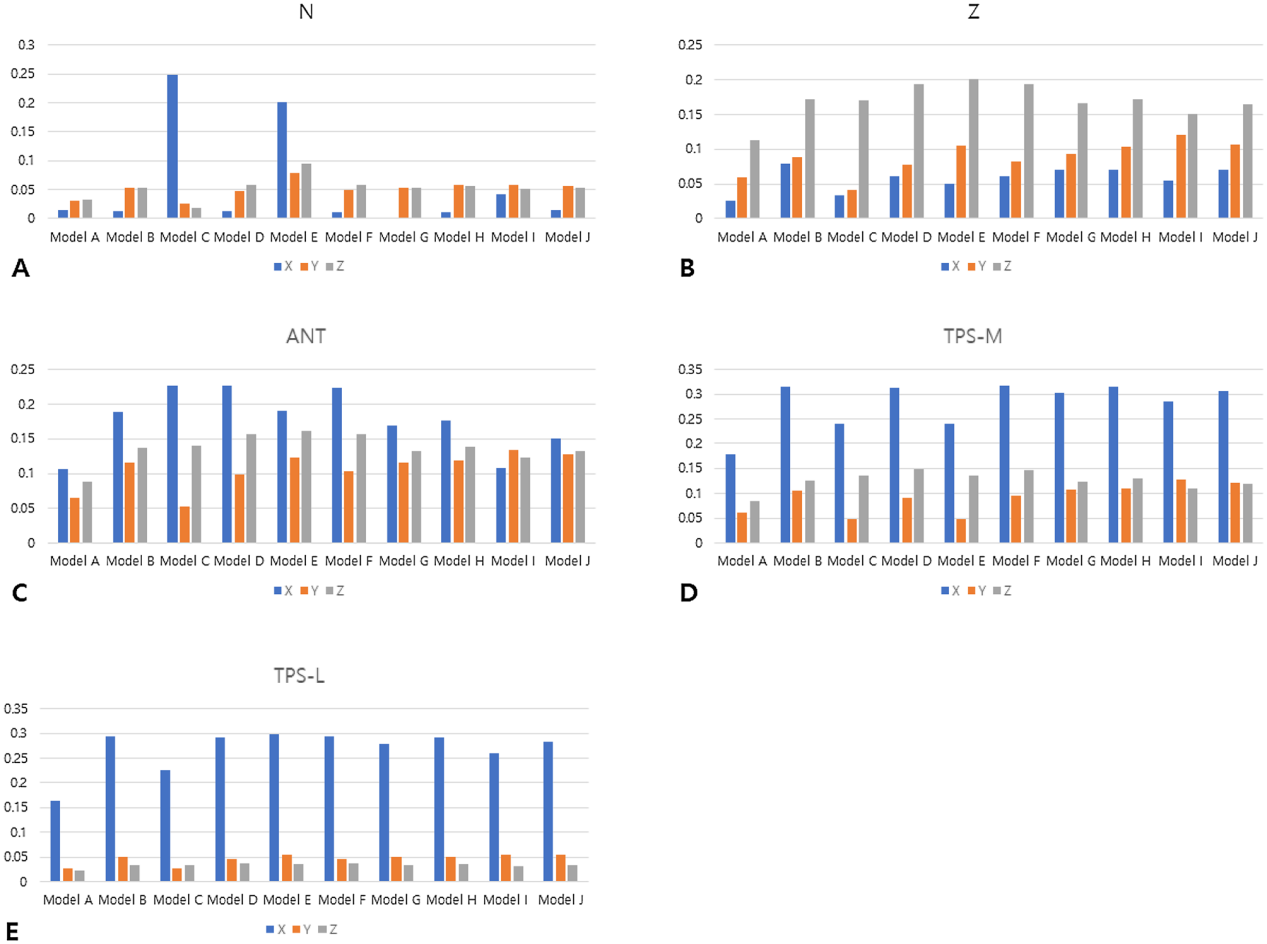
Absolute amount of displacement of each point in the X-, Y-, and Z-axes after palatal expansion. (**A**) N point; (**B**) Z point; (**C**) ANT point; (**D**) TPS-M point; (**E**) TPS-L point. All points experienced displacement in the buccal (X-axis), posterior (Y-axis), and downward (Z-axis) directions.

**Figure 2:**
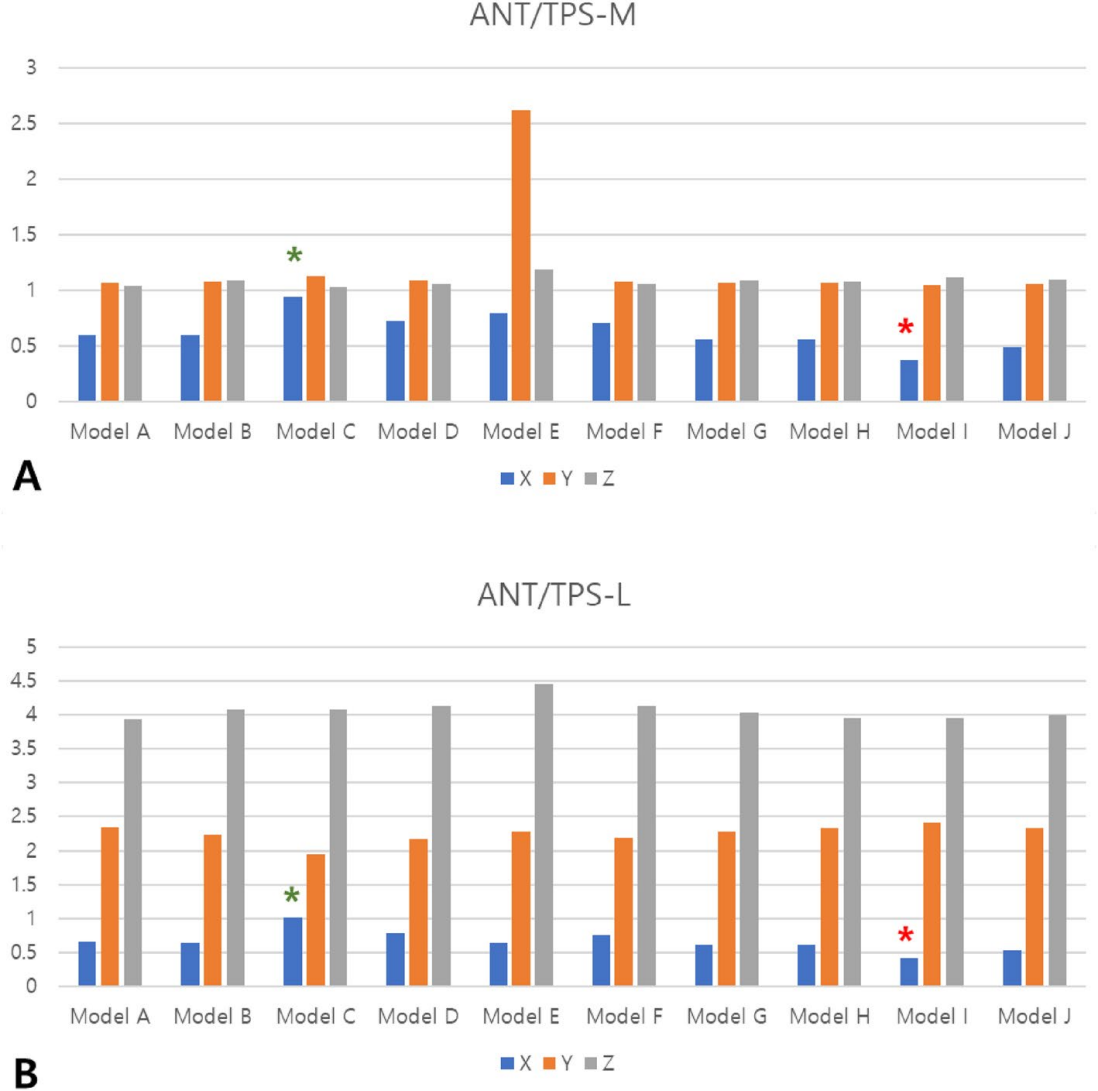
The ratio of ANT point to TPS-M and TPS-L points in the X-, Y-, and Z-axes. (**A**) Ratio of ANT point to TPS-M point; (**B**) Ratio of ANT point to TPS-L point. Green asterisks denote the points with the highest values, while red asterisks indicate the points with the lowest values.

Table 2 presents the displacement amounts in the X-, Y-, and Z-axis directions at points N, Z, ANT, TPS-M, and TPS-L. A positive value along the X-axis indicates movement in the buccal direction, a positive value along the Y-axis indicates movement in the anterior direction, and a positive value along the Z-axis indicates upward movement. In all models, the X-axis values were positive, while the Y-axis and Z-axis values were negative. This means that all points experienced movement in the buccal, posterior, and downward directions. The largest variation in displacement values along the X-axis direction occurred when the expansion screw was activated. Model A, which had the smallest change in molar axis (Table 1), exhibited the smallest displacement amounts at each point (Table 2). Comparing Models C to F and Models G to J as grouped, there was a notable difference in the ANT point, with greater lateral expansion observed in Models C to F where the miniscrews were relatively anteriorly located. The expansion of the TPS-M and TPS-L points was almost identical in both groups. The ratios of ANT/TPS-M and ANT/TPS-L are provided in Table 2, with the largest ratio found in Model C and the smallest ratio in Model I. The displacement amounts and ratios are depicted in Figs. 1 and 2, respectively.

**Table 2:** Amount of displacement of N, Z, ANT, TPS-M, and TPS-L points, and their ratios after palatal expansion in the X-, Y-, and Z-axis directions.

	Axis	Model A (mm)	Model B (mm)	Model C (mm)	Model D (mm)	Model E (mm)	Model F (mm)	Model G (mm)	Model H (mm)	Model I (mm)	Model J (mm)
N	X	0.014558	0.013444	0.248899	0.013400	0.200736	0.010133	0.000557	0.010173	0.041948	0.014325
	Y	− 0.030388	− 0.052761	− 0.024593	− 0.047606	− 0.078754	− 0.049342	− 0.052614	− 0.058073	− 0.057825	− 0.057062
	Z	− 0.031894	− 0.053718	− 0.018073	− 0.058094	− 0.095661	− 0.058448	− 0.052783	− 0.056145	− 0.051405	− 0.053438
Z	X	0.025446	0.078989	0.033444	0.060247	0.050541	0.061583	0.069670	0.069401	0.054819	0.069643
	Y	− 0.059028	− 0.088159	− 0.040500	− 0.077754	− 0.105532	− 0.082050	− 0.093210	− 0.103139	− 0.120313	− 0.106845
	Z	− 0.112238	− 0.171797	− 0.169960	− 0.193254	− 0.200571	− 0.193432	− 0.165796	− 0.171829	− 0.150250	− 0.163848
ANT	X	0.106761	0.189141	0.226405	0.227119	0.190769	0.222948	0.169047	0.176109	0.106938	0.149678
	Y	− 0.064604	− 0.114581	− 0.053442	− 0.098405	− 0.123519	− 0.102347	− 0.115776	− 0.118453	− 0.132830	− 0.127614
	Z	− 0.087991	− 0.136168	− 0.139508	− 0.156376	− 0.161372	− 0.156613	− 0.132567	− 0.137860	− 0.122872	− 0.131315
TPS-M	X	0.177492	0.315059	0.240451	0.313434	0.240451	0.317331	0.301948	0.315175	0.284812	0.307322
	Y	− 0.060540	− 0.106696	− 0.047279	− 0.090481	− 0.047279	− 0.094794	− 0.108046	− 0.110722	− 0.126567	− 0.120227
	Z	− 0.085087	− 0.125226	− 0.135694	− 0.147721	− 0.135694	− 0.147438	− 0.122202	− 0.128340	− 0.110373	− 0.11974
TPS-L	X	0.163607	0.292881	0.224968	0.291238	0.297384	0.294500	0.279463	0.291713	0.260399	0.283368
	Y	− 0.027613	− 0.051198	− 0.027463	− 0.045359	− 0.053981	− 0.046914	− 0.050604	− 0.050677	− 0.055096	− 0.054802
	Z	− 0.022333	− 0.033353	− 0.034108	− 0.037927	− 0.036305	− 0.037939	− 0.032794	− 0.034924	− 0.031134	− 0.032761
ANT/TPS-M	X	0.601496	0.600335	0.941586	0.724614	0.793381	0.702573	0.559857	0.558765	0.375467	0.487041
	Y	1.067134	1.073901	1.130363	1.087568	2.612550	1.079676	1.071543	1.069821	1.049487	1.061440
	Z	1.034131	1.087376	1.028105	1.058585	1.189232	1.062231	1.084821	1.074184	1.113245	1.096662
ANT/TPS-L	X	0.652547	0.645794	1.006388	0.779840	0.641490	0.757040	0.604900	0.603705	0.410668	0.528213
	Y	2.339653	2.238020	1.946009	2.169482	2.288173	2.181573	2.287864	2.337421	2.410887	2.328626
	Z	3.940058	4.082677	4.090218	4.123065	4.444848	4.128065	4.042435	3.947486	3.946565	4.008295

In X-axis, positive value means buccal direction; In Y-axis, positive value means anterior displacement; In Z-axis, positive value means apical direction.

**Figure 3:**
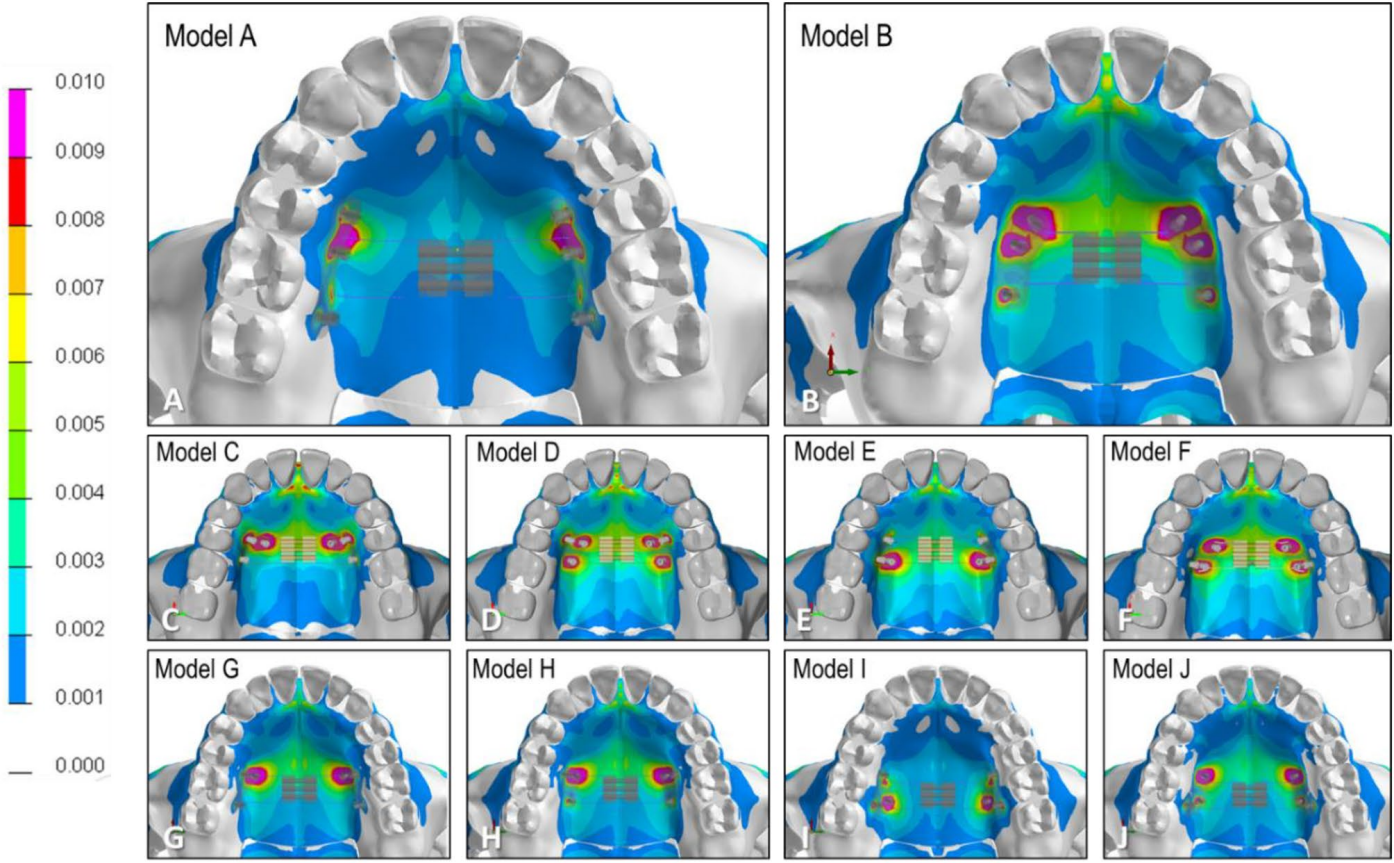
Von Mises stress distribution of the C-expander on each model.

Figure 3 illustrates the distribution of von Mises stress for all the models. The stress was concentrated on the miniscrews, with a tendency for greater concentration on the anterior part compared to the posterior part.

## Discussion

According to Cordasco et al.,^24^ significant dimensional increases were observed in the nasal cavity, particularly in the lower portion, during rapid maxillary expansion. This suggests that the expansion of the nasal floor plays a significant role in the overall volume increase of the nasal cavity. Based on this perspective, it can be inferred that there would be variations in the changes in nasal cavity dimensions and improvements in airway resistance based on the expansion of the palatal bone, which is directly associated with the nasal floor. Additionally, we observed changes by specifically setting the N point at the outermost part of the nasal cavity in the model to better visualize and directly observe the impact on nasal cavity volume changes.

On the contrary, studies conducted by Asanza et al. and Palaisa et al. indicate that the posterior portion of the palate tends to exhibit greater resistance to expansion compared to the anterior portion. This resistance is attributed to the locking effect of the pyramidal processes of the palatal bones into the pterygoid plates of the sphenoid bone.^25,26^ Analyzing the findings of our research, we observed that in Model I, the lateral displacement ratio of ANT to TPS-M and ANT to TPS-L was significantly smaller compared to the other models. This implies that the expansion of the posterior part of the palate was more effective than the anterior part in Model I. Additionally, the N point exhibited the highest amount of lateral displacement in Model I. Collectively, these observations suggest that positioning miniscrews as specified in Model I may be the most effective approach to achieve nasal cavity expansion, particularly in the posterior region.

Furthermore, in the case of Model A, which is the most commonly used conventional miniscrew placement pattern, the lateral changes in the ANT, TPS-M/L, and Z points were the smallest compared to the other models under the same conditions. This implies that Model A can be considered as the least favorable screw placement position for overall expansion of the nasomaxillary complex. The key difference between Model A and the other models lies in the vertical position of the screws, with all six screws being located 7 mm below the CEJ in Model A. When the expansion screw was expanded by the same amount, Model A exhibited significantly less expansion compared to the other models.

From a clinical standpoint, guidelines can be established for selecting the location of miniscrews in orthodontic palatal expansion. If it is necessary to achieve effective expansion in both the anterior and posterior parts of the palate by a similar amount, it is recommended to place miniscrews as demonstrated in Model C, where the values of ANT/TPS-M and ANT/TPS-L in the X-axis direction are close to 1. Model C, which exhibited higher absolute expansion amounts compared to other models, can be considered the optimal miniscrew placement pattern for orthodontic palatal expansion. On the other hand, in the case of Models D and F, the amount of expansion in the anterior part is similar to that of Model C, but the expansion in the posterior part is greater. If it is necessary to expand the anterior part of the palate while simultaneously pursuing more aggressive expansion in the posterior part, miniscrews can be placed as demonstrated in Models D and F. The inverse relationship between the ANT/TPS-M and ANT/TPS-L values and the expansion of the posterior part compared to the anterior part suggests that Model I is the most efficient. However, it is important to note that the decrease in the ANT/TPS-M or ANT/TPS-L values in Model I is due to the reduction in expansion in the anterior part, rather than an increase in the expansion amount in the posterior part. Therefore, it is recommended to utilize Model I only in cases where minimizing expansion in the anterior part is necessary, while considering the shape of the maxillary arch. Model I and J have similar miniscrew positions, but the distribution of stress was more concentrated in the posterior region in Model I compared to Model J. This discrepancy may account for the differences in the ANT/TPS-M and ANT/TPS-L values between the two models.

The change in tooth axis is also an important factor to consider when expanding the palate. One of the main goals of using a bone-borne palatal expander is to minimize the buccal inclination of posterior teeth during expansion.^21,22^ Since the expander is not connected to the teeth, the molars themselves do not experience buccal inclination in the alveolar bone. However, buccal inclination of the posterior teeth may still occur when using a bone-borne palatal expander, as the center of expansion is located in the upper part of the facial bone. In Model I, where the expansion of the nasal cavity was most significant, the buccal inclination of the posterior teeth showed fewer changes compared to the other models. In many cases of maxillary constriction, the upper posterior teeth are buccally inclined as a compensatory mechanism for the narrow palatal width. In such cases, Model I may be ideal for minimizing secondary problems such as periodontal tissue damage or incomplete occlusal formation due to palatal cusp sagging, by reducing the inclination of the posterior teeth. However, it is important to note that placing two miniscrews between the first and second molars, as in Models I and J, can present challenges in terms of difficulty and anatomical structure. Therefore, a cautious approach is required when considering this placement option.

On the contrary, the expansion of the nasomaxillary complex leads to an inevitable increase in the width of the nose and zygomatic area.^27,28^ If avoiding widening of the nose is desired for aesthetic reasons, miniscrews should be placed following the pattern seen in Model G. This placement strategy aims to minimize changes in nasal width while still achieving effective expansion of the posterior part of the palate to some extent. Additionally, some patients may strongly resist any changes to their zygomatic area. In such cases, it is recommended to place the miniscrews in areas where the Z-point shows minimal changes, as seen in Models A and C. In summary, different variations in the miniscrew placement position can be utilized depending on the treatment goals and the specific needs of the patient. The selection should consider factors such as aesthetic preferences, concerns about changes in nasal width or zygomatic area, and the desired extent of posterior palate expansion.

The initial version of the C-expander featured four miniscrews that had undergone surface treatment known as sandblasting with large-grit and acid-etching (SLA). These miniscrews exhibited partial osseointegration with the palatal bone, enabling them to withstand the significant forces generated by the expander. However, due to the strong integration of the miniscrews with the bone, a few miniscrews experienced fracture during the removal process. Subsequently, a revised version of the C-expander was developed, utilizing six miniscrews without surface treatment to prevent fractures. The addition of two extra miniscrews allowed the expander to withstand forces without experiencing fractures, despite having a lower level of osseointegration compared to the SLA-treated miniscrews. The acrylic resin used for the C-expander is polymerized outside the mouth, except for a very small portion around the miniscrew placement sites. Only a small section of polymerization occurs inside the mouth, which does not result in severe inflammation of the mucosa.

When considering the placement of miniscrews, it is important to take into account the anatomical features of the chosen sites. In this study, the miniscrews were positioned either 7 mm or 15 mm below the cementoenamel junction (CEJ) in the premolar area. One critical factor that influences miniscrew placement in this region is the thickness of the soft tissue.^29^ The palatal slope is covered by thick palatal mucosa, and to ensure that the miniscrews do not become buried under the mucosa, it can be beneficial to use miniscrews with longer necks and apply an acrylic pad without delay. Determining the appropriate length of miniscrews and their insertion depth to prevent perforation into the maxillary sinus region can be aided by CT evaluations, which offer insights into the thickness of both bone and soft tissue. Tooth- or gingiva-borne silicone guides can also prove invaluable in guaranteeing the precise placement of miniscrews.^30^ Certain areas, such as the nasopalatine foramen and greater palatine foramen, should be avoided as sites for miniscrew placement. In this study, the selected sites were located at a distance from these foramens. While it is possible for vessels and nerves to traverse the palatal area, the risk of damaging them after miniscrew placement is generally low.^31^ It's noteworthy that the pathway of the greater palatine nerve and artery can often be identified by their distinct coloration and tactile characteristics. The nerve and vessel pathway typically exhibits a bluish color and a softer, more delicate texture, unlike the pink hue and firm sensation characteristic of other areas of the palatal mucosa. Thus, meticulous observation of color and tactile cues can be essential in avoiding any potential damage to these structures during miniscrew placement.

In this study, only the tendency of stress distribution was evaluated, without quantitatively measuring the stress levels. From a clinical perspective, the ability of the miniscrews to withstand the stress generated by the expander is assessed based on the observed stress distribution. However, it is important to note that the specific effect of this stress on the separation of the midpalatal suture could not be evaluated, as the magnitude of the stress was not measured in this study. Understanding the required force to initiate suture separation is crucial, and further research is needed to investigate the relationship between stress levels and suture opening. This would involve studying the stress magnitudes involved and their impact on the midpalatal suture, allowing for a more comprehensive understanding of the effects of stress distribution on suture separation.

In this study, the calculations and analyses were conducted based on the assumption that three miniscrews, which is a common number for C-expander placement, were used per side. However, it is worth noting that the number of miniscrews can be modified, with options of two or four miniscrews. Further research is needed to investigate the potential differences in skeletal changes that may arise depending on the number of miniscrews utilized. Understanding the impact of varying the number of miniscrews on the expansion outcomes and related skeletal changes would contribute to optimizing the treatment approach and expanding our knowledge in this field. Additionally, while this study assumed miniscrew placement at 7 mm and 15 mm below CEJ, it's essential to recognize that the reaction to expansion may vary according to each patient's unique anatomy in a real clinical setting. Nevertheless, the tendencies observed in this study are expected to provide valuable insights into the general response to expansion. Further research is required to explore changes in response to the anatomical variations of the palate in a clinical context.

## Conclusions

The location of the miniscrew used in the tissue- and bone-borne expander had a significant impact on molar inclination and displacement of landmarks on the bone. From the findings of this study, it is recommended to place miniscrews following the pattern seen in Model I to achieve maximum active expansion of the nasal cavity and relative expansion of the posterior portion of the palate compared to the anterior part. For achieving similar amounts of expansion in the anterior and posterior portions of the palate, it is recommended to place a miniscrew as demonstrated in Model C. These results provide valuable insights and pave the way for establishing guidelines regarding miniscrew positioning based on the specific treatment goals. By considering the purpose of treatment, orthodontic practitioners can utilize the findings of this study to optimize miniscrew placement and achieve desired outcomes in palate expansion procedures, although clinical studies should be conducted to support the result of this study.

## Methods

**Figure 4:**
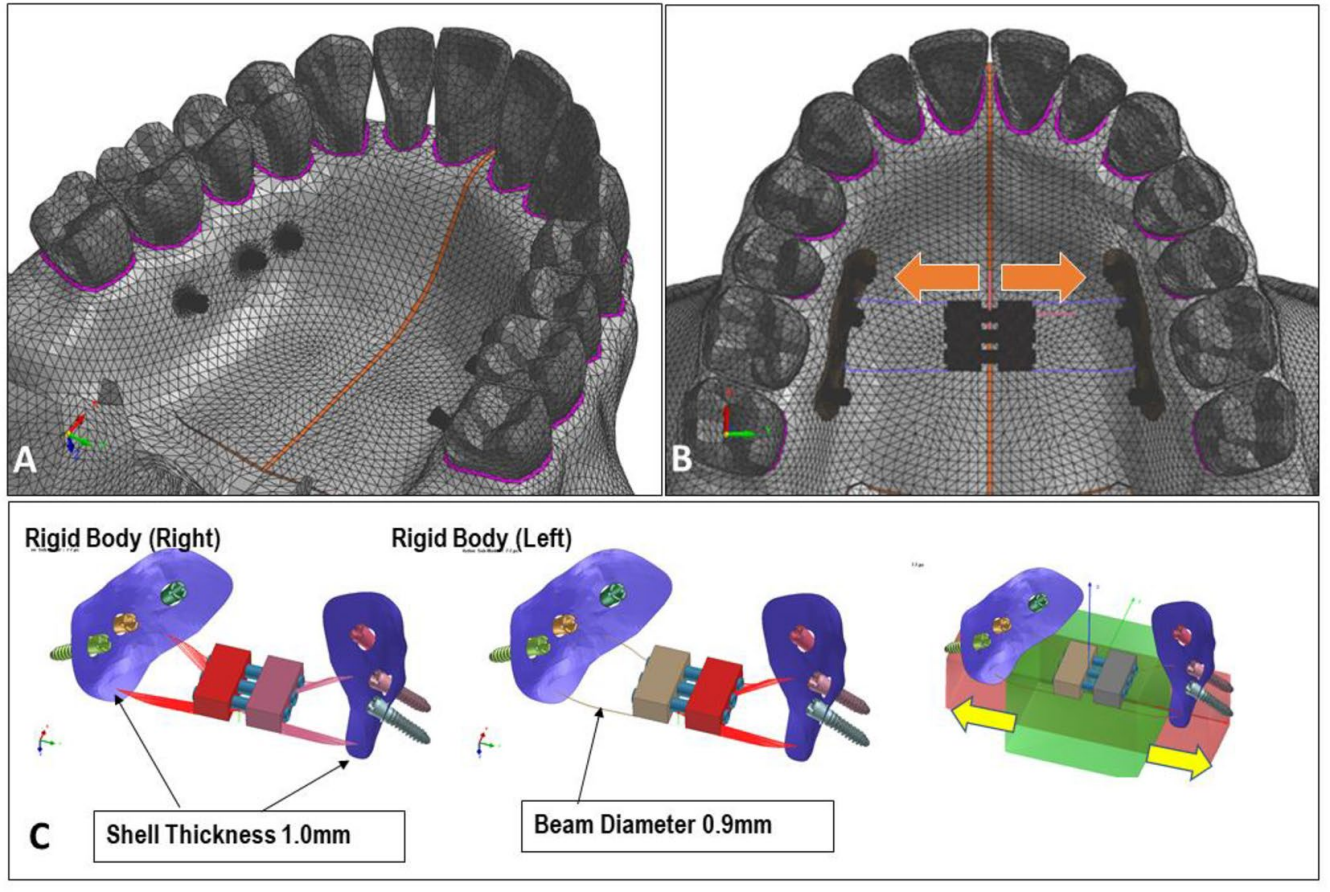
(**A**) Construction of a model composed of tetrahedral finite element mesh; (**B**) Virtual placement of the C-expander, with arrows indicating the direction of expansion; (**C**) C-expander consisting of six miniscrews, resin part, expansion screw, and connecting wire.

The geometric information of a human skull was imported into visual mesh software (version 7.0; ESI Group, Paris, France) to create a tetrahedral finite element (FE) mesh. The model included the maxilla, zygomatic bone, nasal bone, alveolar bone, teeth, and periodontal ligament. The skull model was generated using CT data from an adult male cadaver. To simulate the tissue and bone-borne palatal expander used in clinical practice, a virtual expander was created. It consisted of six miniscrews with a diameter of 1.6 mm and a length of 8.0 mm. The choice of 8.0 mm in length considered the thickness of the palatal mucosa. Bicortical positioning was not necessary as the miniscrews were placed on the palatal slope. The mesh size for the FE model was set at 2.0 mm, except for the area around the miniscrew, which was set at 0.2 mm to capture finer details (Fig. 4). The bone, teeth, and periodontal ligament structures were considered homogenous and isotropic. To replicate the opening pattern of the suture, the midpalatal and nasofrontal sutures were programmed to exhibit elastoplastic behavior. The modulus of elasticity and Poisson's ratio for each structure are provided in Table 3.

**Table 3:** Mechanical properties of each component in the finite element model.

	Young’s modulus (GPa)	Poisson’s ratio
Cortical bone	13.7	0.3
Trabecular bone	1.37	0.3
Dentin	20.7	0.3
Periodontal ligament	0.000168	0.49
Miniscrew (Titanium)	110	0.35

Sutures were modeled to exhibit elastic bilinear behavior based on a previous study. The initial Young's modulus for the sutures was set to 1.0 MPa. A threshold stress (transition) of 0.1 MPa was defined, indicating the point at which the material transitions from elastic to plastic behavior. The final elastic modulus was set at 0.01 MPa, representing the stiffness of the material after the transition.^32^ To establish a fixed reference point, the foramen magnum was set as immobile and served as the origin point in the coordinate system. The coordinates within the model were defined using the transverse plane as the X axis, the sagittal plane as the Y axis, and the vertical plane as the Z axis. This coordinate system allowed for the precise analysis and measurement of displacement and stress distribution within the model.

In the experimental setup, a C-expander was utilized as the tissue and bone-borne expander. The expander consisted of a pad, which included six miniscrews with a diameter of 1.6 mm and a length of 8.0 mm (Bioaction screw; Jin Biomed co., Bucheon, Korea). Additionally, an expansion screw (Forestadent co., Pforzheim, Germany) and a self-polymerization resin (Forestacryl; Forestadent co., Pforzheim, Germany) were incorporated (Fig. 4). The resin pad was designed to cover the oral mucosa and was placed on each side of the expander, where three miniscrews were positioned. This design allowed for the effective transmission of force to the surrounding tissue during the expansion period. The expansion screws were connected to the expander using four connector arms, facilitating the controlled expansion of the appliance.

**Figure 5:**
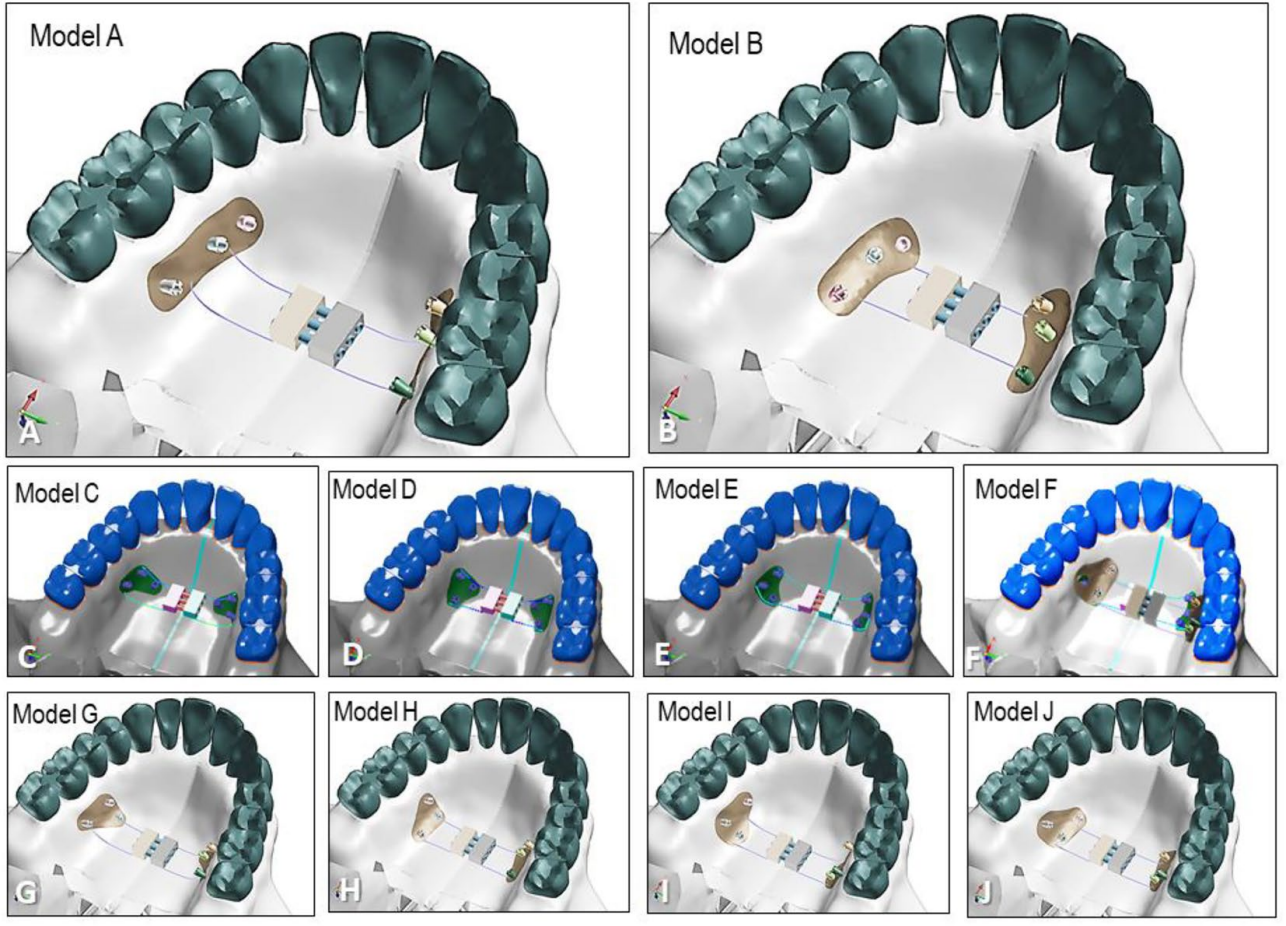
Differential placement of miniscrews in each model. There are ten possible combinations with three miniscrews on each side.

Ten experimental models were created, each with different placement positions for the miniscrews. In Model A, the miniscrews were placed between the roots of the first and second premolars, between the roots of the second premolar and first molar, and between the roots of the first and second molars, all positioned at a point 7 mm below the cemento-enamel junction (CEJ). Model B had the same miniscrew locations as Model A, except they were placed at a point 15 mm below the CEJ. For Models C, D, E, and F, three miniscrews were selectively placed between the first and second premolars and between the second premolar and first molar, at both 7 mm and 15 mm below the CEJ. Similarly, for Models G, H, I, and J, three miniscrews were selectively placed between the second premolar and first molar and between the first and second premolars, also at both 7 mm and 15 mm below the CEJ (Fig. 5). The specific distances of 7 mm and 15 mm below the CEJ were determined based on a previous study for consistent and standardized placement across the models.^23^.

In the study, the expansion screw was uniformly activated at 0.25 mm in the X-axis for all ten models, regardless of the specific expansion force applied. It was assumed that no parts of the expander experienced deformation during the expansion process. A static-geometric nonlinear simulation was conducted using an implicit method implemented in Virtual Performance Solution software (version 2018; ESI Group, Paris, France). The simulations were performed on a computer equipped with an Intel(R) Xeon(R) CPU E5-2680 v4 @ 2.40 GHz with 28 cores and 130 GB RAM, providing sufficient computational power for the analysis.

**Figure 6:**
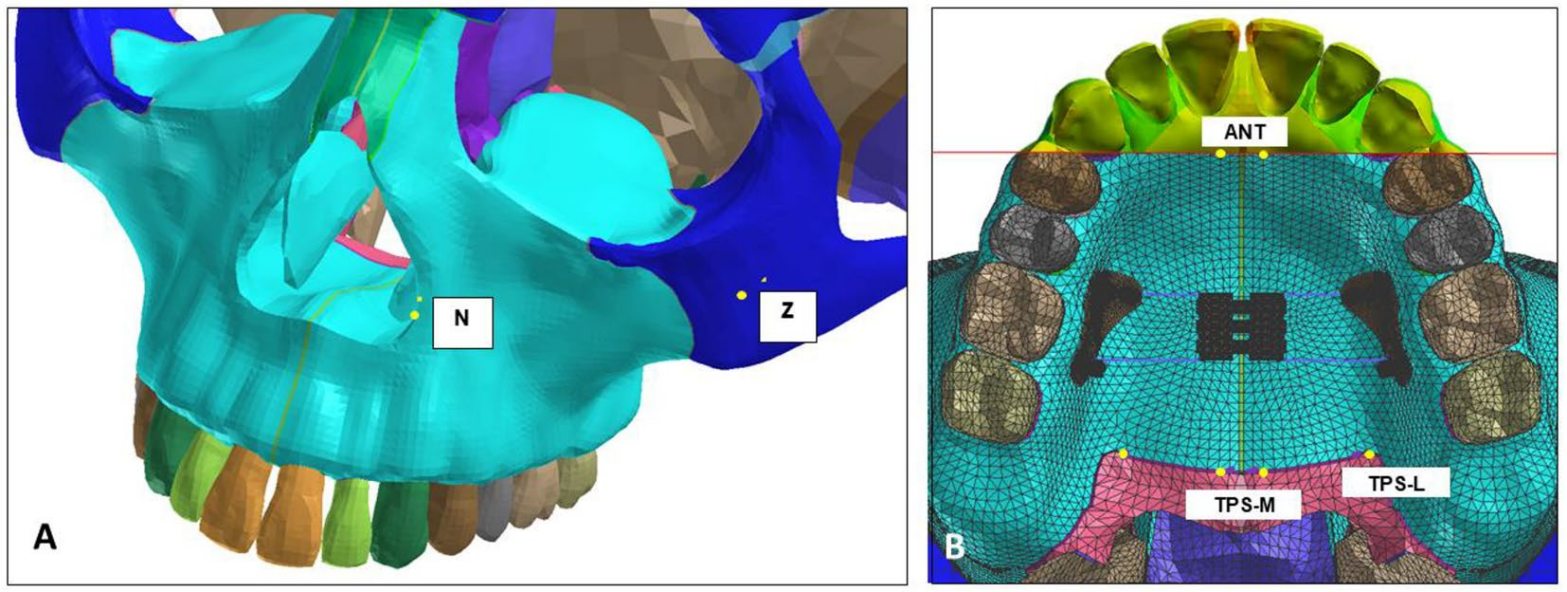
(**A**) Landmarks on the surface of the skull; (**B**) Landmarks on the palatal bone.

To observe detailed changes in the nasomaxillary complex and nasofrontal suture, five specific landmarks were included in the analysis. These landmarks, as depicted in Fig. 6, are as follows:

- Z: The most lateral point of the zygomatic arch in the frontal view.
- N: The most lateral point of the piriform aperture on the maxillary bone surface.
- ANT: A 2 mm lateral point from the midpalatal suture on the line connecting the bilateral upper canines.
- TPS-M: A 2 mm lateral point from the midpalatal suture on the transverse palatal suture.
- TPS-L: The most lateral point of the transverse part on the transverse palatal suture.

These landmarks were selected to capture specific locations and provide a comprehensive evaluation of the changes in the anterior and posterior parts of the palate and the nasomaxillary complex. The ratios of ANT to TPS-M and ANT to TPS-L were calculated to compare the expansion patterns between the anterior and posterior parts of the palate, respectively.

## Data Availability

The datasets generated and/or analysed during the current study are not publicly available due to ethical restriction but are available from the corresponding author (S.H.K.) on reasonable request.
